# Alternative approach of a Fibroepithelial polyp

**DOI:** 10.1590/S1677-5538.IBJU.2016.0471

**Published:** 2017

**Authors:** Cristiano Linck Pazeto, Willy Baccaglini, Thiago Fernandes Negris Lima, Alexandre Gomes S. Simões, Sidney Glina

**Affiliations:** 1 Departamento de Urologia, Faculdade de Medicina do ABC, Santo André, SP, Brasil

## Abstract

A 41-year-old male presented at Emergency Department (ED) with right flank pain associated with hematuria for 3 days. Patient had a previous history of nephrolithiasis. The physical examination and blood tests were normal. Urine analyses showed haematuria > 1.000.000/μL. After clinical evaluation, a computer tomography (CT) showed right ureteral dilatation caused by a 5 mm proximal stone and a distal intraluminal mass of 8 cm in length. In this setting, an ureteroscopic biopsy was performed and revealed a large polypoid lesion histologically suggestive of fibroepithelial polyp. Due to technical difficulties (intraluminal mass length and technical issue for the passage of guidewire) and after discussing all available minimally invasive options, we opted for a laparoscopic approach. Instead of ureterectomy of the affected segment of the ureter, as classically performed, we proceeded with an ureterotomy, blunt dissection of the tumor and ureterolithotomy, with complete removal of the mass. This approach did not require ureteral anastomosis and the ureteral dilatation facilitated its primary closure. No complications occurred, even after 3 years of follow-up.

## ARTICLE INFO

**Available at: http://www.intbrazjurol.com.br/video-section/20160471-pazeto_et_al/**

**Int Braz J Urol. 2017; 43 (Video #19): 1195-1195**

